# Death from Ether

**Published:** 1877-01

**Authors:** 


*Death from Ether.*—From the daily press we take the following, satisfied that the administration of the agent was carefully and scientifically performed, and that no blame can be justly charged against the operator:

"Walter Lewis, aged 12, died in a dentist's chair at Rahway, N. J., Friday evening, in fifteen minutes after taking ether in order to have a tooth extracted. Dr. Westlake, the dentist, is prominent in his profession. The death is supposed to have resulted from an irregulation of the heart."

